# Protective role of CFTR during fungal infection of cystic fibrosis bronchial epithelial cells with *Aspergillus fumigatus*

**DOI:** 10.3389/fcimb.2023.1196581

**Published:** 2023-08-23

**Authors:** Beate Illek, Horst Fischer, Terry E. Machen, Gopika Hari, Karl V. Clemons, Gabriele Sass, Jose A. G. Ferreira, David A. Stevens

**Affiliations:** ^1^ UCSF Benioff Children's Hospital Oakland, Children's Hospital Oakland Research Institute, Oakland, CA, United States; ^2^ Department of Molecular and Cell Biology, University of California, Berkeley, Berkeley, CA, United States; ^3^ California Institute for Medical Research, San Jose, CA, United States; ^4^ Division of Infectious Diseases and Geographic Medicine, Stanford University Medical School, Stanford, CA, United States

**Keywords:** *A. fumigatus conidia*, *A. fumigatus germlings*, *A. fumigatus hyphae*, gliotoxin, cystic fibrosis, CFTR ion channel, zona occludens protein 1

## Abstract

Lung infection with the fungus *Aspergillus fumigatus (*Af*)* is a common complication in cystic fibrosis (CF) and is associated with loss of pulmonary function. We established a fungal epithelial co-culture model to examine the impact of Af infection on CF bronchial epithelial barrier function using Af strains 10AF and AF293-GFP, and the CFBE41o- cell line homozygous for the F508del mutation with (CF^+CFTR^) and without (CF) normal CFTR expression. Following exposure of the epithelial surface to Af conidia, formation of germlings (early stages of fungal growth) was detected after 9-12 hours and hyphae (mature fungal growth) after 12-24 hours. During fungal morphogenesis, bronchial epithelial cells showed signs of damage including rounding, and partial detachment after 24 hours. Fluorescently labeled conidia were internalized after 6 hours and more internalized conidia were observed in CF compared to CF^+CFTR^ cells. Infection of the apical surface with 10AF conidia, germlings, or hyphae was performed to determine growth stage-specific effects on tight junction protein zona occludens protein 1 (ZO-1) expression and transepithelial electrical resistance (TER). In response to infection with conidia or germlings, epithelial barrier function degraded time-dependently (based on ZO-1 immunofluorescence and TER) with a delayed onset in CF^+CFTR^ cell monolayers and required viable fungi and apical application. Infection with hyphae caused an earlier onset and faster rate of decline in TER compared to conidia and germlings. Gliotoxin, a major Af virulence factor, caused a rapid decline in TER and induced a transient chloride secretory response in CF^+CFTR^ but not CF cells. Our findings suggest growth and internalization of Af result in deleterious effects on bronchial epithelial barrier function that occurred more rapidly in the absence of CFTR. Bronchial epithelial barrier breakdown was time-dependent and morphotype-specific and mimicked by acute administration of gliotoxin. Our study also suggests a protective role for CFTR by turning on CFTR-dependent chloride transport in response to gliotoxin, a mechanism that will support mucociliary clearance, and could delay the loss of epithelial integrity during fungal development *in vivo*.

## Introduction

Human airways are exposed to *Aspergillus fumigatus (A. fumigatus)*, a mold pathogen, by daily inhalation of hundreds of conidia, which are readily cleared by healthy people (Hohl and Feldmesser, 2007; Bigot et al., 2020). However, pulmonary infections caused by *A. fumigatus* are a major cause of morbidity and mortality globally (Latge and Chamilos, 2019). Compromised lung defenses arising from immunosuppression, chronic respiratory conditions, and concomitant viral or bacterial pulmonary infections are recognized risks factors for the development of pulmonary aspergillosis (Huang et al., 2022; Earle et al., 2023). *A. fumigatus* is implicated in up to 90% of all cases with pulmonary aspergillosis (Perfect et al., 2001) and is the most common and clinically significant fungus isolated from cystic fibrosis patients (Warris et al., 2019). In people with cystic fibrosis, repeated episodes of infection and airway inflammation are associated with deterioration of lung function. The ability of *A. fumigatus* to adhere to and internalize in lung epithelial cells (the initial step in airway invasion) has been studied, and *in vitro* and *in vivo* models were developed in order to elucidate the pathobiology of *Aspergillus*-related disease states (Dagenais and Keller, 2009; Latge and Chamilos, 2019).

Mutations of the cystic fibrosis transmembrane conductance regulator (CFTR) gene leading to CF affect the function of several organs and tissues, particularly the bronchial epithelial cells. Lung epithelial CFTR dysfunction leads to dysregulation of epithelial electrolyte flux and build-up of hyperviscous mucus, increasing risk of respiratory bacterial and fungal infection (Gadsby et al., 2006; Kreda et al., 2012). As people with CF are living longer as a result of therapeutic advances, and as intensive antibiotic suppressive therapy for bacterial infection becomes more common, the incidence and prevalence of *A. fumigatus* infection has increased over the past decades (Bargon et al., 1999; Valenza et al., 2008). The onset of highly effective CFTR modulator therapy has improved pulmonary function and mucociliary clearance in many CF patients, however, beneficial outcomes on fungal infections remain to be seen (Bercusson et al., 2021; Chesnay et al., 2022). The effect of colonization or infection with *A. fumigatus* on CF lung function at the cellular level is incompletely understood. Investigations have suggested that the fungus can elicit aberrant pulmonary inflammation in the setting of CFTR mutations (Chotirmall et al., 2013; Burgel et al., 2016). The interaction between *A. fumigatus* and the tight junctions (TJ) of CF respiratory epithelium has received relatively little study despite the relevance of *A. fumigatus* in CF lung disease.

The integrity of the epithelium in the upper and lower respiratory tract and the proper functioning of ciliated epithelium are essential for clearance of conidia and prevention of fungal infection (Hohl and Feldmesser, 2007; Segal, 2009; Ben-Ami et al., 2010). One overlooked component of epithelial defenses in lungs are apical junctional complexes that form between neighboring cells and consist of TJ. TJ form an apical barrier, regulate paracellular transport of ions and water, and maintain planar tissue architecture for smooth mucus flow and mucociliary clearance (Anderson, 2001; Ganesan et al., 2013). TJ are formed by not only the integral membrane proteins but also by many peripheral membrane proteins (PMP). One of the major TJ’s PMP is the zonula occludens 1 (ZO-1), a cytoplasmic-facing protein that is critical for tight-junction stability and linkage of the complex to the actin cytoskeleton (Zahraoui et al., 2000). Transepithelial resistance studies demonstrate the ability of specific fungal aeroallergens and mycotoxins to cause deterioration of the airway epithelial barrier and tight junction integrity (Botterel et al., 2002; Khoufache et al., 2007; Zaidman et al., 2017). Dysfunction of epithelial junctions and alteration of normal ZO-1 protein distribution are increasingly linked to airway diseases and may predispose to infections; however, a comprehensive picture of the relationship between junctional integrity and fungal colonization or infection is lacking.

In this investigation we studied the roles of *A. fumigatus* at different growth stages (conidia, germlings, and hyphae) and its virulence factor gliotoxin to identify if the epithelial integrity and the distribution of the TJ protein, ZO-1, are differentially impacted. We hypothesize that the comparison between the effects of these different morphotypes over the TJ structure could point to some of the features that make *A. fumigatus* destructive to CF lungs. We explored the role of F508del mutant vs. normal CFTR function by examining responses in CF vs. CFTR-corrected CF bronchial epithelial cell monolayers, respectively.

## Materials and methods

### Epithelial cell culture

The bronchial epithelial cell line CFBE41o- homozygous for the F508del mutation was used as a model CF airway epithelium and referred to as CF cells in this study. Complemented CF cells (referred to as CF^+CFTR^), stably expressing an Epstein-Barr virus (EBV)-based episomal expression vector, pCEP4β (InVitrogen, Carlsbad, CA) containing 6.2 kb full-length wtCFTR cDNA (Illek et al., 2008) were used to compare responses to *A. fumigatus* infection in CF and CFTR-corrected CF cells. The CF and CF^+CFTR^ bronchial epithelial cell lines were maintained in Eagle’s Minimum Essential Media (MEM) cell culture medium (Gibco, Life Technologies, Grand Islands, NY) supplemented with 10% fetal bovine serum (FBS), penicillin (100 U/ml), streptomycin (100 µg/ml), and glutamine (2 mM) at 37°C in a humidified 5% CO_2_, 95% air incubator. Cells grown to 80% confluence were treated with 0.25% trypsin-0.1% ethylene glycol-bis (p-aminoethyl ether)- N,N,N’,N’-tetra acetic acid solution for 5-15 min. Cell suspensions were counted and seeded directly onto Transwell® permeable filter supports (0.45-µm pore size, 12-mm diameter; Corning Inc., NY) at a density of ~10^5^ cells/insert. All inserts were pre-coated with a mixture of 0.01 mg/ml human fibronectin (BD Biosciences, San Jose, CA), 0.029 mg/ml Vitrogen 100 collagen (Cohesion Technologies, Palo Alto, CA), and 0.1 mg/ml bovine serum albumin (Biosource/Biofluids, Camarillo, CA) overnight. A total of 500 µl and 1000 µl of fresh media were added to the upper and lower compartment of the Transwell insert, respectively.

### Fungal culture

*Aspergillus fumigatus* conidia, strain 10AF (Denning et al., 1990) and green fluorescent protein (GFP) expressing Af293 (Nierman et al., 2005) were obtained as follows: *A. fumigatus* was taken from stock suspensions stored at -80°C and then grown for 4 days on Potato Dextrose Agar (Becton Dickinson and Co., Sparks, MD) at 37°C. Plates containing the GFP-Af293 were protected from light. Four-day-old conidia were harvested by gently washing with 0.05% Tween-80 (J.T. Baker Chemical Co., Phillipsburg, NJ) in 0.9% saline (Baxter Healthcare Corp., Deerfield, IL).

### Light-microscopic evaluation of fungal growth on bronchial epithelial cells

One sterile 10-mm cover slip (Roundcover10; BIPEE, Shenzhen, Guangdong, China) was placed in each well of two 24-well plates. CF, or CF^+CFTR^ cells (10^5^ cells/ml) were seeded in one 24 well plate, respectively, in a volume of 1 ml MEM/10% FBS with penicillin (100 U/ml) and streptomycin (100 µg/ml) (P/S). Twenty-four hours after seeding, the medium in each well was replaced by one ml of MEM with 10% FBS with P/S to remove non-attached cells. Cells were allowed to grow to confluency, with medium changes as needed. When cells reached confluency, medium was replaced by 1 ml MEM/10% FBS with PS, containing 10^4^ 10AF conidia. To document with images, cover slips were removed from their wells and placed face down on glass microscope slides (12-544-7; Fisher Scientific, Waltham, MA). Pictures were taken using a digital microscope (M83EZ-C02; OMAX Microscopes, China), in combination with OMAX Toup View Software. Growth of conidia were analyzed at 0, 3, 6, 9, 12, and 24 hours after infection at a magnification of 400x. At each timepoint multiple pictures of two replicate setups were taken. Representative pictures are shown.

### GFP-expressing Af293 conidia binding assay

The binding assay was modified from that of Chaudhary (Chaudhary et al., 2012). CF and CF^+CFTR^ cells were plated in quadruplicate on Transwell® culture inserts as described above and grown to confluence. For the conidia challenge, inserts containing the cell cultures on the apical side were washed three times with sterile MEM and over­laid with ~10^4^ GFP-expressing Af293 conidia suspension in MEM. Mixtures were incubated at 37°C and 5% CO_2_ protected from light. After 6 h, wells were washed three times with PBS and incubated with 25 mM Calcofluor White (Molecular Probes, Eugene, OR) in ice-cold PBS for 10 minutes. Filters from Transwell inserts were cut out and placed on a glass-bottom dish for confocal imaging. GFP fluorescence was excited with a 488 nm laser and Calcofluor White was excited with a 405 nm laser.

### An *in vitro* coculture model of *A. fumigatus* on CF and CF^+CFTR^ cells

**Figure 1:**
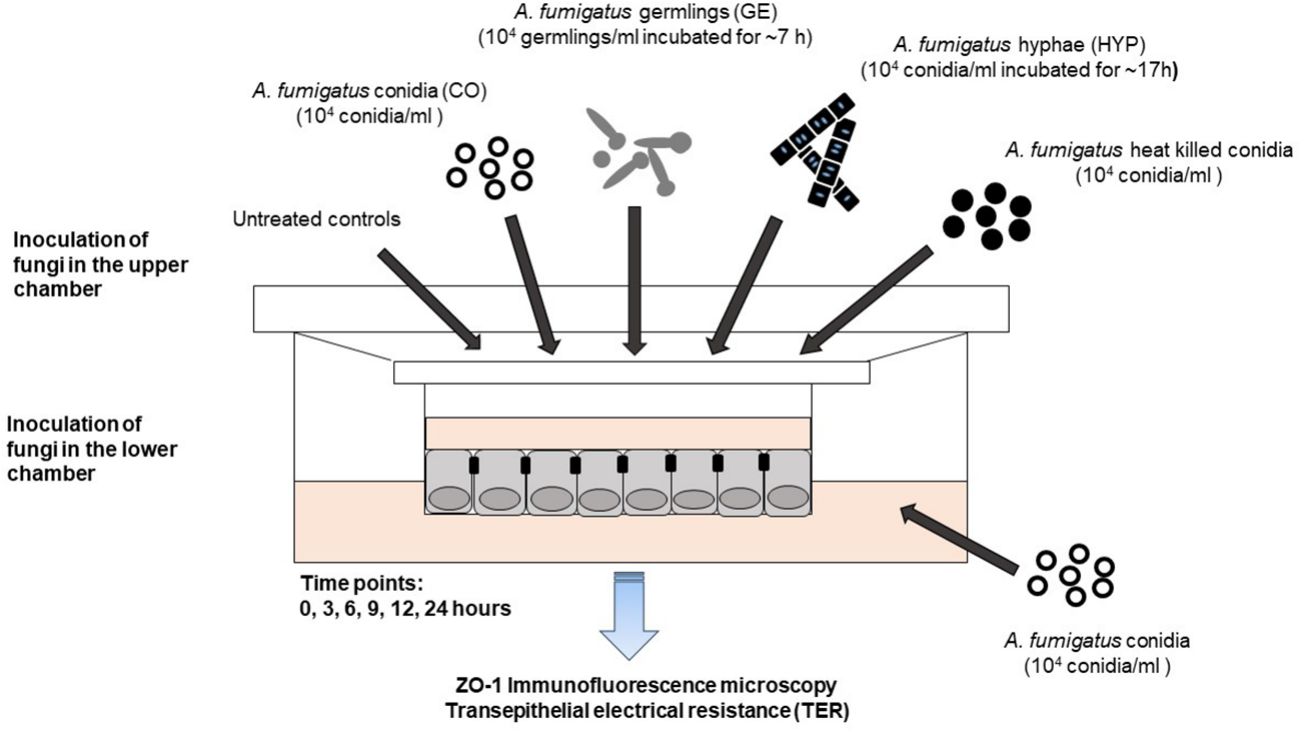
Fungal epithelial co-culture model. Development of a bronchial epithelial co-culture model to study the impact of *A. fumigatus* infection on cystic fibrosis (CF) cells. Planar 2-dimensional (2D) bronchial epithelial cultures with and without normal CFTR protein expression were exposed to different morphotypes of *A. fumigatus*. *A. fumigatus* conidia (CO), germlings (GE), or hyphae (HYP) were added to the apical surface (top) of the cell monolayer at a final concentration of 5,000 CO, GE, or HYP per ~10^6^ epithelial cells. Acute effects of gliotoxin, a major *A. fumigatus* virulence factor, were determined at 10 μM gliotoxin and administered to the apical surface. The potency of non-viable *A. fumigatus* conidia was determined by exposing the apical surface to heat-killed Af. conidia for 24 hours. Side-specific effects of *A. fumigatus* conidia were determined by adding Af. conidia to the serosal (bottom) compartment for 24 hours.

CF and CF^+CFTR^ cells were separately grown to confluency on Transwell® culture inserts, as described above and used for experiments when the transepithelial electrical resistance (TER) was above 800 Ω×cm^2^ (Illek et al., 2010). Prior to the fungal challenge the MEM cell culture medium was aspirated from the apical side of the Transwell® inserts. The different fungal suspensions were prepared as follows. The conidia (CO) in the suspension were adjusted to a final concentration of ~10^4^/mL in MEM + 10% FBS. Germlings (GE) were obtained after ~7 h of *A. fumigatus* incubation in sterile MEM +10% FBS at 37°C and 100 rpm shaking. Hyphal suspension (HYP) was prepared from a 10^4^ conidia/ml suspension (5 mL MEM cell culture medium supplemented with 10% FBS) that was incubated with gentle agitation (100 rpm) at 37°C for ~18 h. Development of the germlings into hyphae was considered mature after 50% of the germinated conidia presented at least one septum; the resulting hyphal suspension (500 µl) was used in the experiments. *A. fumigatus* CO, GE or HYP were added to the apical side of the insert. In separate experiments, CO was added to the basal side of the cell monolayers at 5000 CO per ~10^6^ cells (**Figure 1**).

### Conidia heat treatment

*A. fumigatus* CO were heat-killed (CO_HT_) by incubation at 65°C for 1 h and their viability was assessed on Sabouraud Dextrose Agar (Gersuk et al., 2006). The treatment was repeated until no viable conidia were detected on Sabouraud agar plates. Conidia were kept at −20°C between heat-treatments. *A. fumigatus* heat-killed CO suspensions were centrifuged, washed three times, and suspended in MEM +10% FBS before use. *A. fumigatus* heat-treated CO were added to the apical side of the cell monolayers at final concentration of 5000 CO/~10^6^ cells.

### Transepithelial electrical resistance measurements

Epithelial and tight junction integrity of confluent CF and CF^+CFTR^ cell monolayers was assessed by measuring the TER with an epithelial volt-ohm-meter (EVOM, Millicell-ERS, Darmstadt, Germany) (Srinivasan et al., 2015). TER (reported as Ω.cm^2^) was measured at 0, 3, 6, 9, 12, and 24 h after apical exposure to *A. fumigatus* CO, GE, or HYP. Assays were performed with n=4 or n=8 replicates per group in 2-3 independent experiments for *A. fumigatus* CO, GE, and HYP.

### Ussing chamber studies

A serosal-to-mucosal Cl gradient was applied to measure acute effects of gliotoxin on gradient driven Cl currents and loss of transepithelial resistance as previously described (Illek et al., 2008). For measurements of transepithelial Cl current, CF and CF^+CFTR^ cell monolayers were grown on Snapwell inserts (Corning, Corning Inc., NY, USA) and mounted into water-jacketed Easy Mount Ussing chambers (Physiologic Instruments, San Diego, CA). Transepithelial voltage was clamped to 0 mV and resulting short-circuit current (I_sc_) was measured using a four-electrode voltage clamp (VCC MC6 multichannel voltage clamp, Physiologic Instruments, with Ag-AgCl electrodes (World Precision Instruments, Sarasota, FL) connected to the solutions through agar bridges (3%) containing 1 M KCl. Positive currents were defined as chloride anion movement from serosa to mucosa. I_SC_ was recorded on a computer (DI-710, DataQ Instruments, Akron, OH). Transepithelial voltage was clamped from 0 to 1 mV for one second every minute. TER was calculated off-line from the corresponding current deflections using Ohm’s law. Values were analyzed at 10, 30-, 60-, 90-, and 180-min time points.

The composition of the serosal high Cl solution was (in mM): 120 NaCl, 25 NaHCO_3_, 5 KCl, 1.2 NaH_2_PO_4_, 5.6 glucose, 1.0 CaCl_2_, and 1.2 MgCl_2_. The mucosal solution was Cl-free and had the following composition: 120 sodium gluconate, 25 NaHCO_3_, 5 potassium gluconate, 1.2 NaH_2_PO_4_, 5.6 glucose, 2.5 calcium gluconate_2_, and 1.2 magnesium gluconate. Both mucosal and serosal solutions were continuously gassed with 95% O_2_/5% CO_2_, resulting in pH 7.4. Ussing chambers were kept at 37°C with a temperature-controlled water bath circulator.

Inhibitors were used to evaluate the effects of gliotoxin on transcellular vs. paracellular chloride transport. The involvement of CFTR was probed by comparing responses to gliotoxin between CF vs. CF^+CFTR^ cell monolayers. Furosemide, an inhibitor of the basolateral Na-K-2Cl (NKCC) cotransporter, was used to determine the involvement of uptake of chloride across the basolateral cell membrane in both CF and CF^+CFTR^ cell monolayers. The presence of an inhibitory effect on short-circuit current (I_Cl_) by furosemide indicates transcellular chloride movement. Gliotoxin (Sigma-Aldrich, St. Louis, Mo.) was prepared as a 10 mM stock solution in DMSO and used at a final concentration of 10 μM. Furosemide (Sigma-Aldrich, St. Louis, MO) was prepared as a 100 mM stock solution and used at a final concentration of 100-500 μM.

### Immunofluorescence staining of CF and CF^+CFTR^ cell lines

After 0, 6 and 12 hours of exposure to the fungus (*A. fumigatus* CO, GE or HYP), inserts containing CF or CF^+CFTR^ (treated and untreated controls, for each) were rinsed three times with phosphate buffered saline (PBS pH 7.4, Thermo Fisher, Waltham, MA), fixed for 10 min with 2% formaldehyde in PBS, rinsed with PBS, and permeabilized with 0.5% Triton X-100 (Sigma -Aldrich, St. Louis, MO) in PBS for 15 min. Non-specific binding was blocked by treatment with 1% bovine serum albumin (BSA)-5% goat serum-PBS for 60 min prior to the addition of monoclonal mouse anti-ZO-1 antibody (BD Transduction Laboratories, San Jose, CA). The ZO-1 antibody was incubated for 2 h at room temperature, and then cells were thoroughly rinsed with 1% BSA-PBS and 0.1% BSA-PBS. Finally, cells were incubated for 2 h with Alexa 594-labeled goat anti-mouse IgG secondary antibody (Molecular Probes, Eugene, OR) and for an additional 10 min with 1 µM Hoechst 33342. Images were obtained on a laser scanning confocal microscope (Zeiss LSM710) using a x63/1.4 numerical aperture oil objective (Carl Zeiss, Thornwood, NY). Samples were excited at 405 nm and 561 nm laser light. Some fluorescent images were overlaid onto the corresponding bright-field images. For the final display, all images were lightened by ~20%.

### Statistical methods

Statistical analysis was performed using SigmaPlot (version 15, Systat Software, San Jose, CA). Dependent on the outcome of tests for normality (Shapiro-Wilk) and equal variances (Brown-Forsythe) nonparametric tests were applied. Determination of statistical significance within one treatment group was performed on normalized TER readings obtained from non-treated controls at t=0 or controls at corresponding time points by means of Tukey Test following Repeated Measures ANOVA on Ranks. Determination of statistical significance within two treatment groups was performed at the same time point by means of Welch’s t-test or Mann Whitney Rank Sum. Determination of statistical significance of paired data sets was performed by Wilcoxon Signed Rank Test, a nonparametric equivalent of the parametric paired t-test. Internalization of conidia was tested using a Chi-squared test on data in a contingency table. Data are shown as mean values ± standard deviation (SD); n is the number of experimental runs. *P <*0.05 was considered significant.

## Results

### Development of a fungal CF bronchial epithelial co-culture model

**Figure 2:**
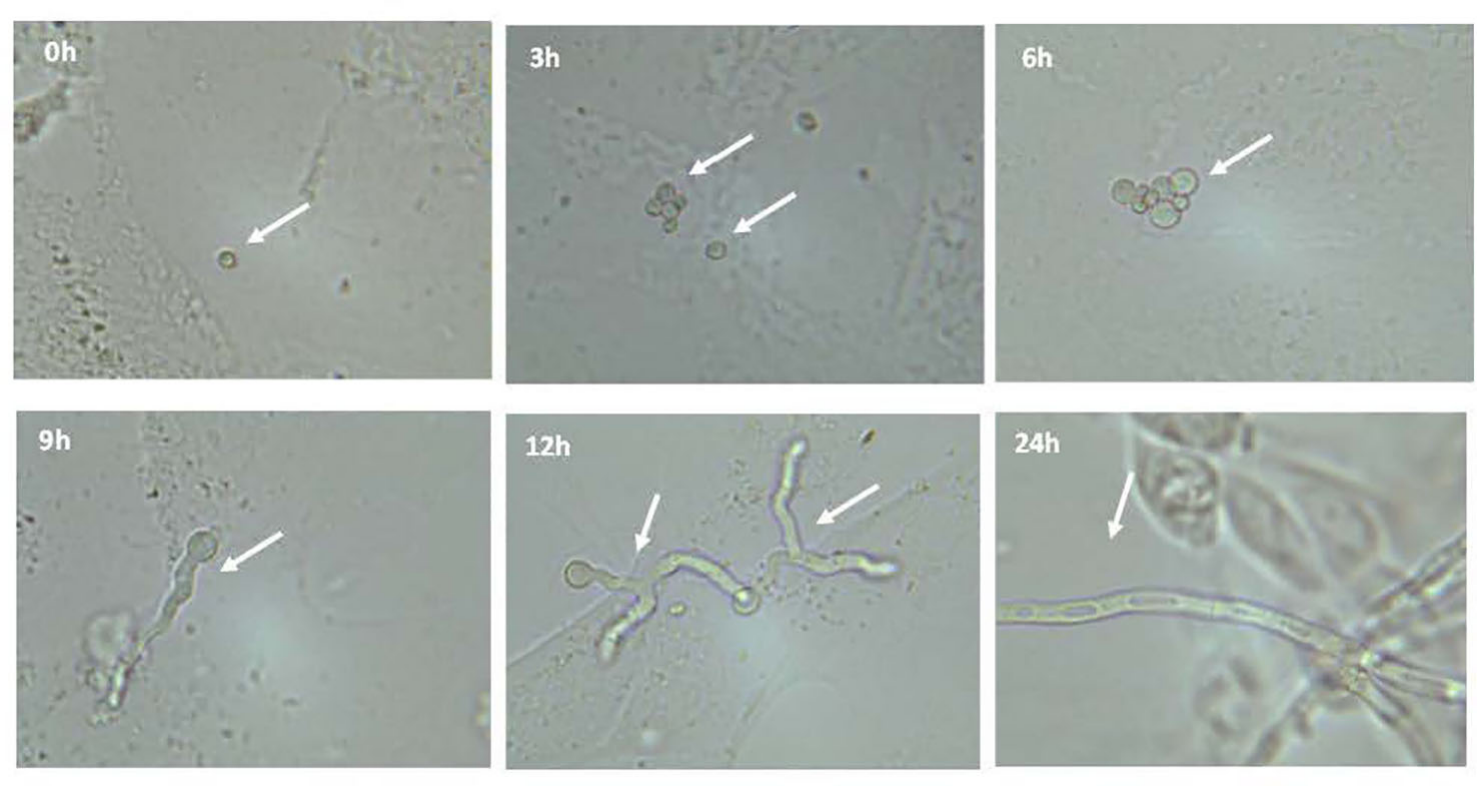
Microscopic observation of growth of conidia from *Aspergillus fumigatus* in presence of bronchial epithelial cells. Confluent CF cells were infected with *A. fumigatus* conidia (strain 10AF). Conidial growth was observed after 0, 3, 6, 9, 12, and 24 hours after infection at a magnification of 400x. Representative images are shown.

A co-culture model was developed to study the impact of fungal infections by *A. fumigatus* on cystic fibrosis bronchial epithelial cells. First, we observed the growth of conidia from *A. fumigatus* in the presence of bronchial epithelial cells. Confluent CF bronchial epithelial cells were infected with *A. fumigatus* conidia (strain 10AF; 5,000 conidia/well) and light microscopy was performed at 0, 3, 6, 9, 12, and 24 hours after infection. Conidia remained unchanged within 3 hours of infection but swelled after 6 hours and grew into germlings within 9 hours of infection. After 12-24 hours of infection, we observed hyphae on cells, with increasing branching and length growth over time. Fungal growth was equivalent on CF^+CFTR^ cells (data not shown). After 24 hours of infection epithelial cells became rounded and partially detached (**Figure 2**), consistent with cell damage.

### Conidia are internalized by CF bronchial epithelial cells

**Figure 3:**
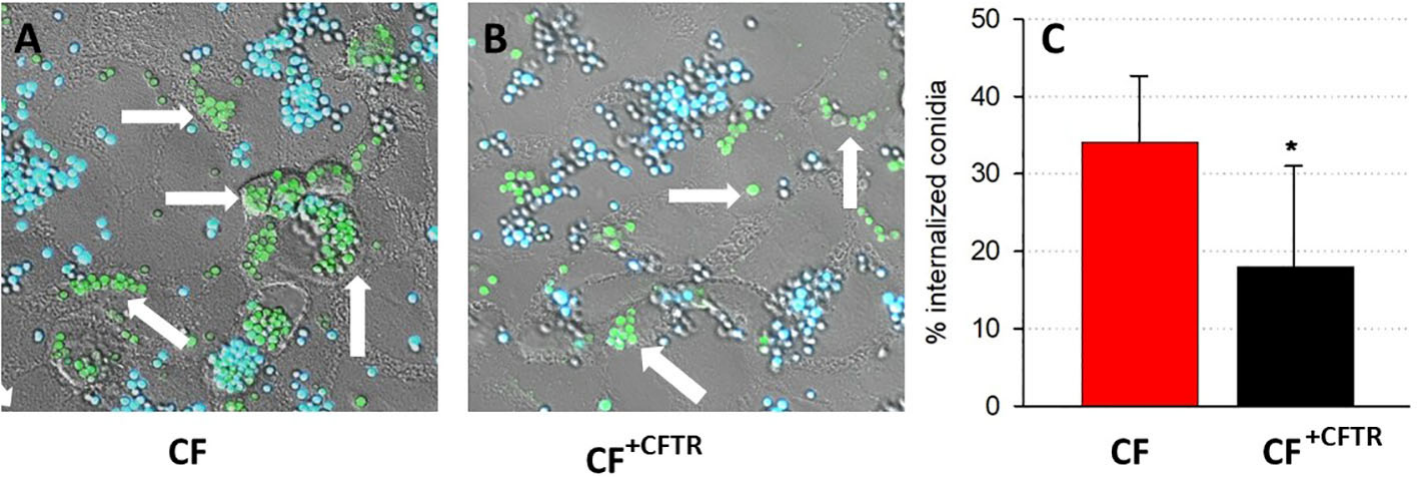
Internalization of conidia in CFBE41o- bronchial epithelial cells. Cellular uptake of GFP-labeled conidia (GFP-Af293; green) is shown in confluent **(A)** CF vs. **(B)** CFTR-corrected (CF^+CFTR^) bronchial epithelial cells after six hours of infection. Calcofluor White counterstaining of GFP-expressing conidia distinguishes between internalized conidia (green, arrow) and external conidia (blue). **(C)** Relative amount of internalized conidia, mean ± SD; significantly different, Chi squared test, p=0.01. Triplicate wells were prepared for each cell type and six random fields were examined to identify internalized and external conidia. Note that the internalization of conidia was reduced in the cytosol of CF^+CFTR^ cells.

The cellular uptake of conidia was determined by exposing CF and CF^+CFTR^ cell monolayers to green fluorescent protein-expressing *A. fumigatus* (GFP-Af293). The cell-impermeant Calcofluor White, which stains extracellular fungal cells blue, was used to differentiate between intracellular (green) and extracellular (blue) conidia in/on the epithelial cells. Both the CF (**Figure 3A**) and CF^+CFTR^ (**Figure 3B**) bronchial epithelial cells internalized a fraction of the GFP-Af293 conidia. After 6 hours of incubation with GFP-Af293 (**Figure 3C**), 35.1 ± 7.2% of conidia were internalized in CF vs. 18.0 ± 13.0% in CF+CFTR cells (significantly different, p=0.01).

### *A. fumigatus* morphotypes cause redistribution of tight junction ZO-1 protein and modification of cellular morphology; effect of CFTR

**Figure 4:**
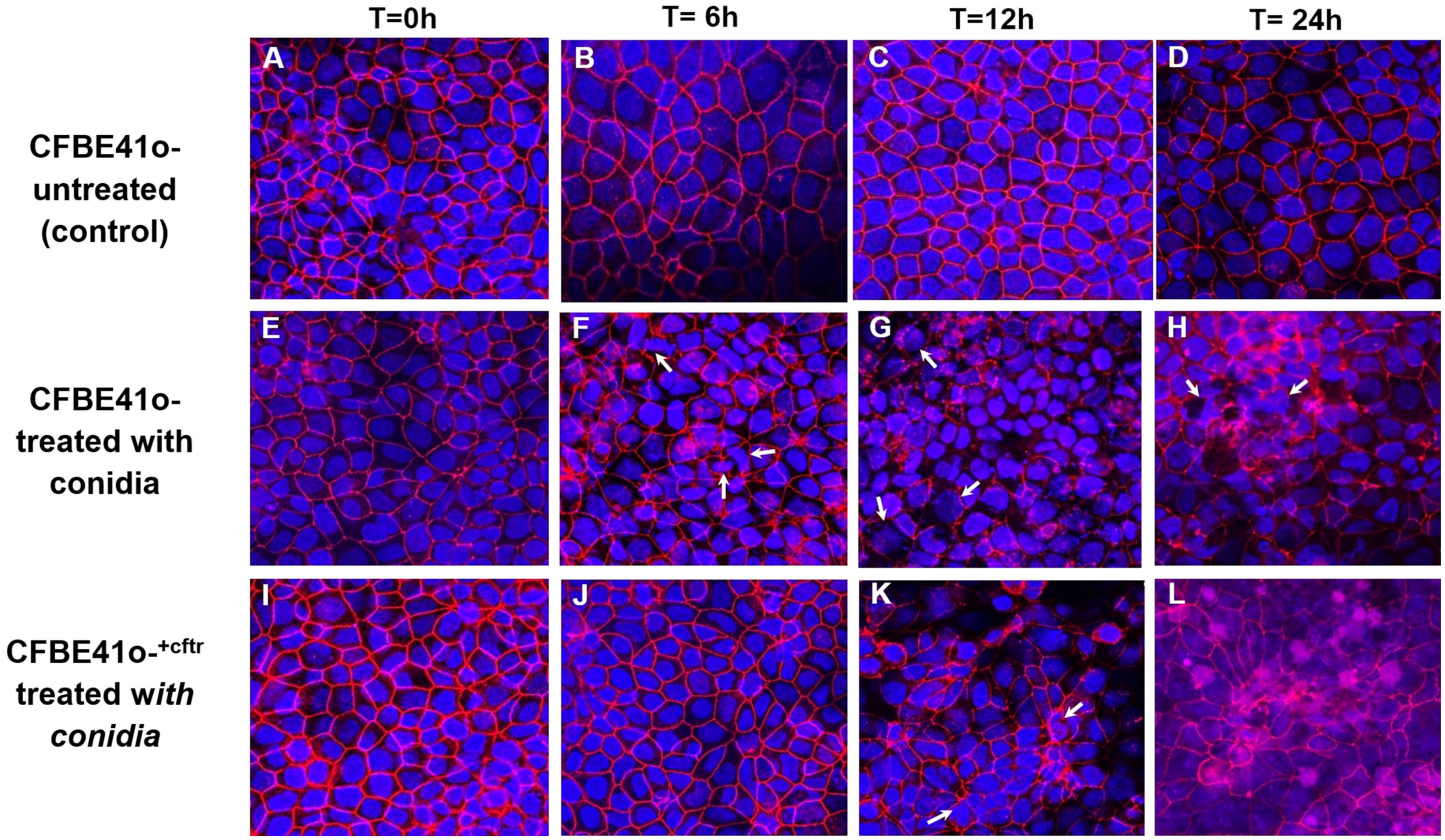
Effect of infection with Af. conidia on epithelial ZO-1 expression. Conidia (CO) cause time-dependent breakdown of apical tight junction protein zona occludens-1 protein (ZO-1) in confluent CF cells and is delayed by CFTR expression in CF^+CFTR^ cells as emphasized at 6 hours post infection. **(A–D)** untreated time controls of CF cell monolayers at time = 0, 6, 12, and 24 hours. **(E–H)** CF cell monolayer treated with CO. After 6 hours of treatment, monolayers showed onset of disorganized ZO-1 immunofluorescence, and the presence of cells containing fragmented or enlarged nuclei (white arrows). Enlarged cells containing fragmented nuclei were also noted after 24-hour treatment with CO (white arrows). **(I–L)** CF^+CFTR^ cell monolayer treated with CO. Images represent typical fields of view and are typical for one of two similar experiments. Blue, nuclei; red, ZO-1; each frame is 100 μm wide.

**Figure 5:**
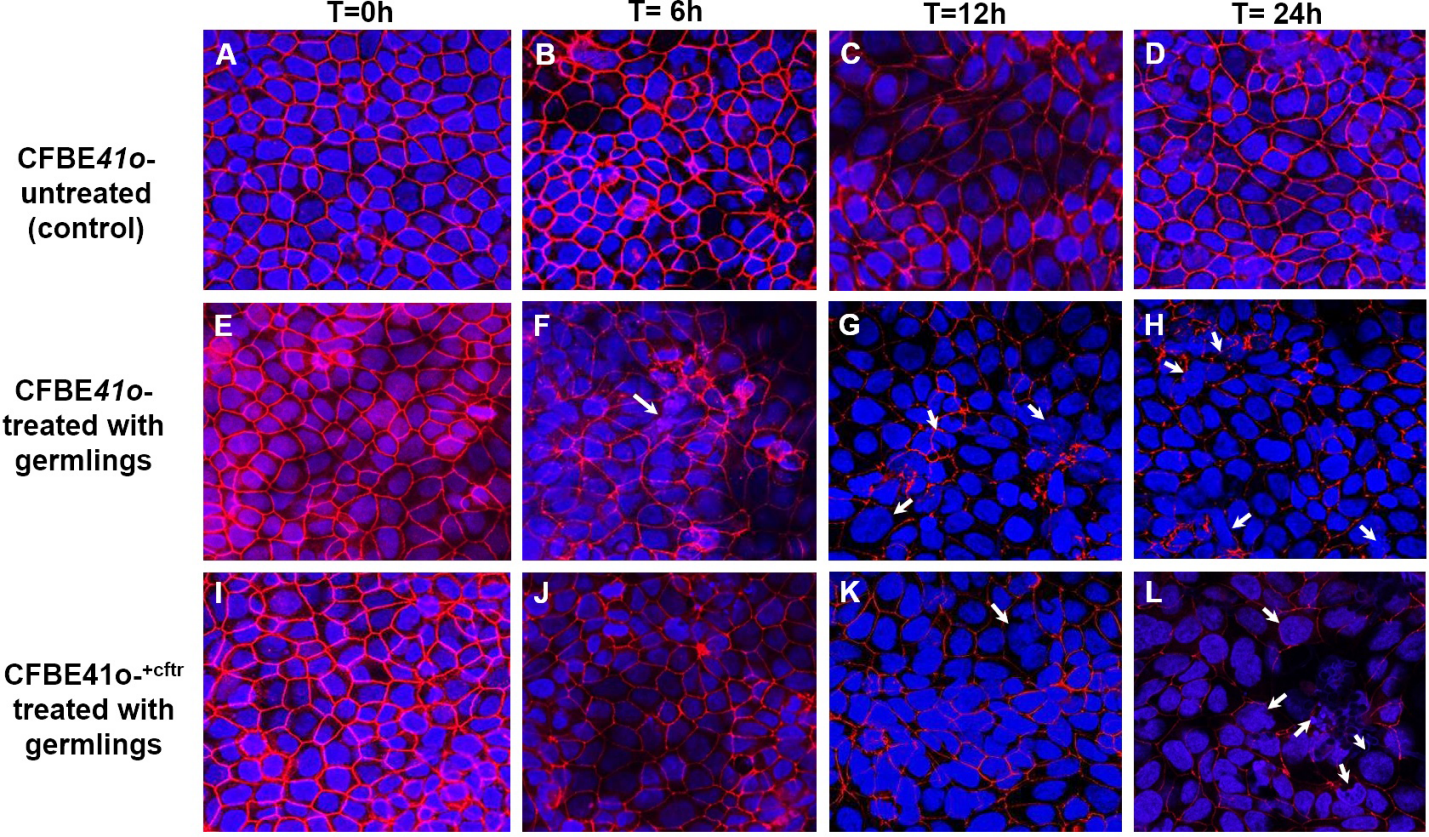
Effect of infection with Af. germlings on epithelial ZO-1 expression. Germlings (GE) cause time-dependent breakdown of apical tight junction protein zona occludens-1 protein (ZO-1) in CF cells and is delayed by CFTR expression in CF^+CFTR^ monolayers as emphasized at 6 hours post infection. **(A–D)** untreated time controls of CFBE41o- epithelia at time = 0, 6, 12, and 24 hours. **(E–H)** CF cell monolayers treated with GE. The presence of enlarged cells containing fragmented or enlarged nuclei (white arrow) was noted after 12-hour **(G)** and 24-hour **(H)** treatment. **(I–L)** CF^+CFTR^ cell monolayers treated with CO. Images are typical fields of view from one of two similar experiments. Blue, nuclei; red, ZO-1; each frame is 100 μm wide.

For morphological observations of fungal induced changes in epithelial tight-junctions, the associated protein zona occludens-1 (ZO-1) was analyzed after infection with Af. conidia (**Figures 4A–L**) or Af. germlings (**Figures 5A–L**). In non-infected, control CF or CF^+CFTR^ cell monolayers, ZO-1 showed characteristic belt-like immunostaining around the smooth, outer-most perimeters of cells, and nuclei were large and regularly shaped at all time-points (**Figures 4A–D**, **5A–D**). Infection of the CF cell monolayers with CO (**Figure 4F**) or GE (**Figure 5F**) led to a diffuse and disorganized ZO-1 appearance after 6 hours. In contrast, CF^+CFTR^ cell monolayers infected with CO (**Figure 4J**), or GE (**Figure 5J**) for 6 hours appeared similar to uninfected monolayers. However, after 12 hours, infection of CF^+CFTR^ cell monolayers with CO (**Figure 4K**) or GE (**Figure 5K**) induced significant disorganization and, in areas, a total lack of ZO-1 between cells that was similar when compared to CF cell monolayers. These results indicated that the expression of normal CFTR in CF cells delayed the onset of the breakdown of the tight junction protein ZO-1. In terms of morphotype-specific effects, GE-infected cell monolayers (CF: **Figure 5G**; CF^+CFTR^: **Figure 5K**) showed more pronounced loss of ZO-1 tight junction protein than CO infection (CF: **Figure 4H**; CF^+CFTR^: **Figure 4K**) suggesting the GE were more aggressive than CO for the disruption of ZO-1. In some cases, many nuclei were fragmented or enlarged (white arrows). After 24 hours, the growth into hyphae was associated with significant disorganization in both CF and CF^+CFTR^ cells and, in areas, a total lack of ZO-1 between cells, and abnormal nuclei.

### *A. fumigatus* morphotypes and gliotoxin cause time-dependent decrease of transepithelial electrical resistance; effect of CFTR

**Figure 6:**
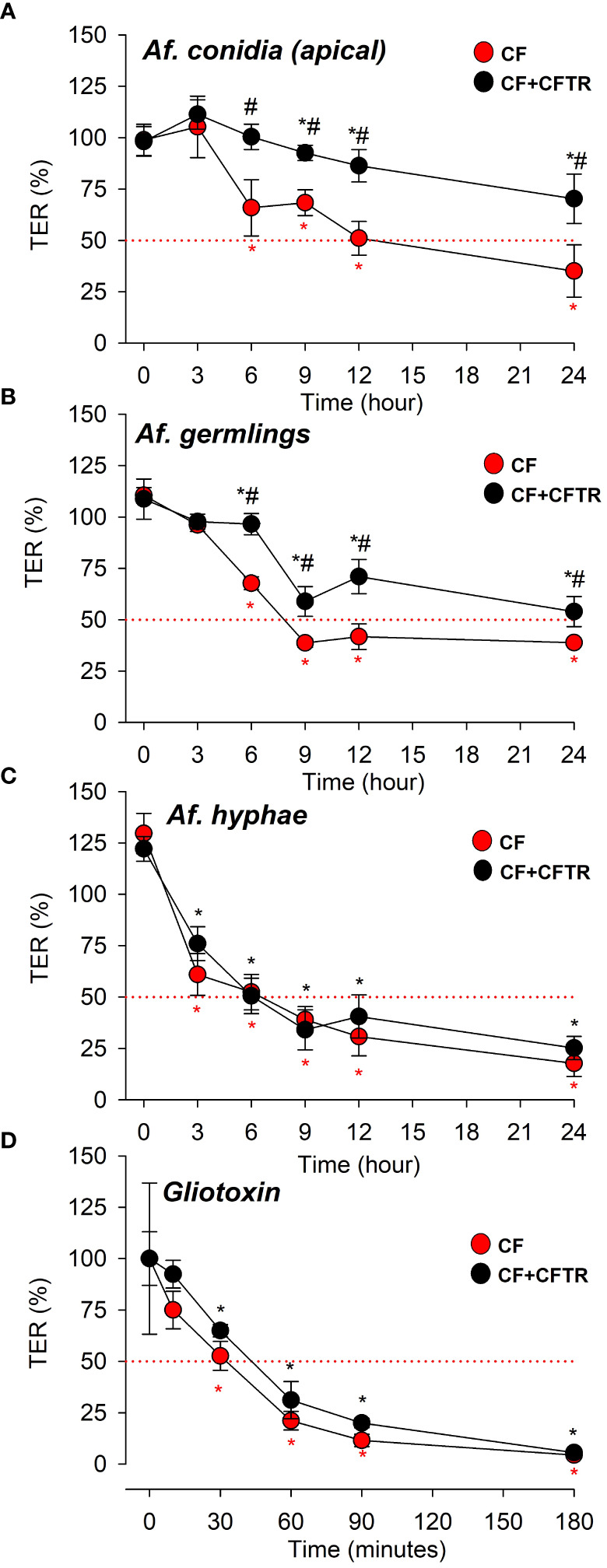
Effects of *A. fumigatus* morphotypes (Af. conidia, Af. germlings, or Af. hyphae) and *A. fumigatus* derived virulence factor gliotoxin on transepithelial electrical resistance (TER). Time-dependent decline of TER values in CF (red circles) and CF^+CFTR^ (black circles) in response to apical exposure to Af. conidia, Af. germlings, Af. hyphae or gliotoxin. **(A)** Af. conidia (5,000 CO/10^6^ cells) decreased TER after t = 6 hours in CF (red asterisk) and t = 9 hours in CF^+CFTR^ (black asterisk) when compared to untreated controls at t=0. Note that TER values were significantly larger in CF^+CFTR^ at t = 6, 9, 12, and 24 hours when compared to CF. TER declined to 50% after 12 hours in CF and remained above 50% after 24 hours in CF^+CFTR^. **(B)** Af. germlings decreased TER after t = 3 hours in CF (red asterisk) and t = 6 hours in CF^+CFTR^ (black asterisk) when compared to untreated controls at t=0. TER values were significantly larger in CF^+CFTR^ at t = 6, 9, 12, and 24 hours compared to CF at same time points. TER declined to 50% after 9 hours in CF and after 24 hours in CF^+CFTR^. **(C)** Hyphae decreased TER after t = 3 hours in both CF (red asterisk) and CF^+CFTR^ (black asterisk) when compared to untreated controls at t=0. Note that TER was similar in CF^+CFTR^ when pairwise compared to CF at same time points. TER declined to 50% after 6 hours in both CF and CF^+CFTR^. **(D)** Gliotoxin (10 μM) decreased TER after t = 30 minutes in both CF (red asterisk) and CF^+CFTR^ (black asterisk) when compared to untreated controls at t=0. Note that TER was similar in CF^+CFTR^ when pairwise compared to CF at same time points. TER declined to 50% after 30-45 minutes in both CF and CF^+CFTR^. TER measurements were performed with the EVOM meter assay **(A‒C)** or with the Ussing assay **(D)**. TER values were normalized to corresponding non-treated time controls **(A‒C)** or before treatment **(D)**. Red dotted line indicates TER at 50% as a reference. Data are shown as mean values ± SD and were obtained from 1-3 independent experiments in each group with 3-8 replicates per time point. Significantly different from untreated controls at t=0 in CF (*, red asterix) or CF^+CFTR^ (*, black asterix) with p<0.05 (Tukey Test following Repeated Measures ANOVA on Ranks). # indicates significant difference between CF and CF^+CFTR^ at the same time point (p<0.05, Welch’s t-test or Mann Whitney Rank Sum).

Confluent CF and CF^+CFTR^ cell monolayers were incubated with CO (**Figure 6A**), GE (**Figure 6B**) or HYP (**Figure 6C**) from the apical side and TER was monitored for 24 hours. After 3 hours, neither CO (**Figure 6A**) nor GE (**Figure 6B**), had significant effect on TER in either CF or CF^+CFTR^ cell monolayers (i.e., when compared to untreated controls at t=0 (p>0.05) or when the readings were compared between CF and CF^+CFTR^ cell monolayers (p>0.05). After 6 hours, both CO (**Figure 6A**) and GE (**Figure 6B**) caused a decline in TER in CF cell monolayers that was 41% and 39% of the control value, respectively (p <0.05). In contrast, CO (**Figure 6A**) or GE (**Figure 6B**) did not affect TER of CF^+CFTR^ cell monolayers at the 6-hour time point but caused TER to decline by 17% (CO) and 36% (GE) of the control value at 9 hours (p<0.05). At 12- and 24-hours post infection, TER had dramatically deteriorated in both CF (CO 56% and 75%, GE 64% and 80%, respectively) and CF^+CFTR^ (CO 23% and 45%, GE 37% and 56%, respectively). Pairwise comparison of TER values in response to CO (**Figure 6A**) or GE (**Figure 6B**) demonstrated that TER significantly declined less in CF^+CFTR^ when compared to CF at t = 6, 9, 12, and 24 hours (indicated as #, p<0.05) suggesting a protective role of CFTR expression.

Treatment with HYP resulted in a significant decline in TER at the early 3-hour time point readings in both CF and CF^+CFTR^ cell monolayers compared to the untreated controls (**Figure 6C**, p<0.05). However, the HYP-induced decline in TER was similar at each time point when the cell lines were compared (p>0.05). Exposure of CF and CF^+CFTR^ cell monolayers to heat-killed conidia (CO_HT_) on the apical cell surface did not cause significant changes of the TER readings compared to the baseline measurements at t=0 over the 24-hour time course of the measurements (**Supplementary Material, Figure S1**). To determine if the signaling mechanisms that lead to the breakdown of epithelia integrity were specifically induced by contact with the apical membrane, a CO inoculum was added to the serosal compartment (basal side) of the cell cultures. No changes on epithelial TER were observed over a 24 h incubation period, in either cell line (**Supplementary Material, Figure S2**).

**Figure 7:**
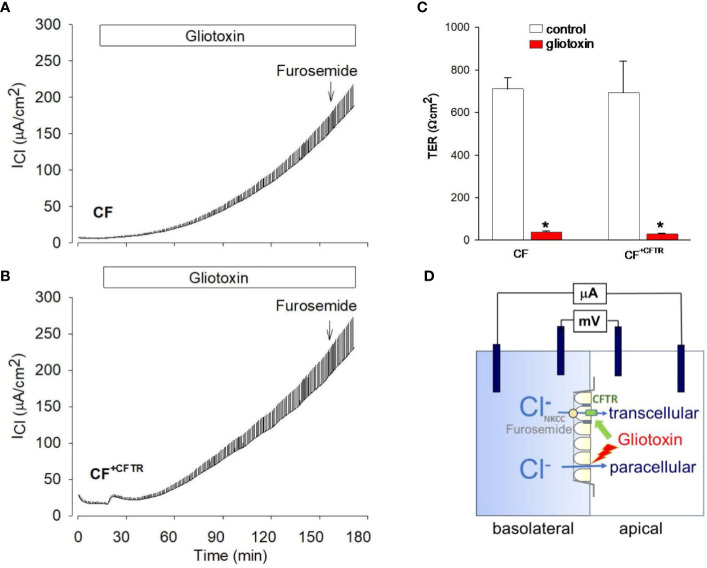
Effect of gliotoxin on chloride current and transepithelial resistance in Ussing chamber experiments. Original recordings of gradient driven chloride currents (I_Cl_) across **(A)** CF cell monolayers or **(B)** CF^+CFTR^ cell monolayers. Current deflections are shown in response to 1 mV voltage pulses and were used to calculate corresponding transepithelial resistances (TER). Note that the height of the current deflections visualizes the changes in transepithelial resistance in response to gliotoxin, i.e., an increase in the size of the current deflection indicates a decrease in transepithelial resistance. After 3 hours, furosemide (500 μM) was added to the serosal compartment to probe for the involvement of cellular chloride uptake across the basolateral cell membrane. Addition of furosemide demonstrated no inhibitory effect on the gliotoxin-activated I_Cl_ suggesting the movement of chloride across the paracellular and not transcellular pathway. **(C)** Summary of TER values before and after gliotoxin treatment for 2-3 hours. Data are shown as mean values ± SE, n=3, * indicates p<0.05 (Wilcoxon Signed Rank Test). **(D)** Epithelial cell model to study the effects of gliotoxin on epithelial barrier function by using a basolateral to apical Cl gradient. Transcellular chloride movement is known as entry of chloride across the basolateral membrane via the Na-K-2Cl (NKCC) cotransporter and exit of chloride ions across the apical membrane via CFTR anion channels. Effective block by furosemide, an inhibitor of NKCC, indicates movement of chloride across the cell. Lack of inhibition by furosemide indicates movement of chloride across the paracellular pathway.

Because gliotoxin is a major *A. fumigatus*-derived virulence factor we examined the possibility of its involvement during the loss of TER (**Figure 6D**). TER was quantified by measuring changes in gradient-driven chloride currents with the Ussing chamber assay. Original data traces of the time course of transcellular chloride currents in response to gliotoxin are illustrated in **Figure 7A** for CF and in **Figure 7B** for CF^+CFTR^ cell monolayers. Exposure of CF cell monolayers to gliotoxin caused a rapid decline in TER within 3 hours, with some initial difference in favor of CF^+CFTR^ cells. For CF cell monolayers, TER declined to 75.0 ± 9%, 52.6 ± 7%, and 21.1% ± 5.0% (p<0.05) at 10-, 30-, and 60-minutes post treatment, respectively. For CF^+CFTR^ cell monolayers, TER declined to 92.4 ± 6.8%, 64.9 ± 3.0%, and 31.1% ± 9.0% (p<0.05) at 10-, 30-, and 60-minutes post-treatment, respectively. By the 3-hour timepoint, TER had drastically declined by 94% in CF cells and by 96% in CF^+CFTR^ cells. A decline of TER by 50% (illustrated by red line in **Figure 6D**) was reached after 35-45 minutes. On average, TER decreased from 793 ± 125 Ω.cm2 (before gliotoxin) to 33.3 Ω.cm^2^ (after 3 hours of gliotoxin) in CF and from 693 ± 144 Ω.cm2 (before gliotoxin) to 30.6 ± 7.6 Ω.cm^2^ in CF^+CFTR^ cell layers (**Figure 7C**). These experiments demonstrated that gliotoxin exposure elicited a rapid decline in TER at a rate that was faster than the rate observed after exposure to *A. fumigatus* hyphae.

At the end of the experiment, furosemide was added to probe for the involvement of cellular chloride uptake across the basolateral cell membrane via the NKCC transporter (illustrated in **Figure 7D**). Addition of furosemide (500 μM) had no inhibitory effect on the gliotoxin-activated I_Cl_ suggesting the movement of chloride across the paracellular and not transcellular pathway.

### Effect of gliotoxin on CFTR-dependent chloride transport

**Figure 8:**
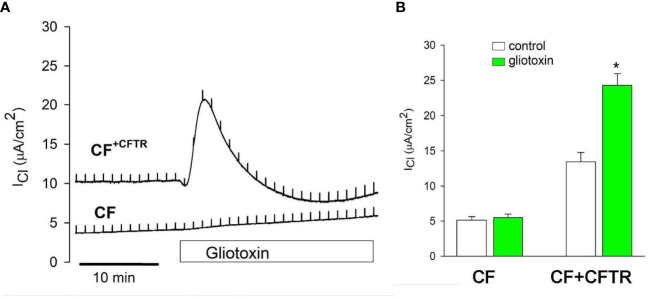
Effect of gliotoxin on CFTR-mediated chloride current. **(A)** Addition of gliotoxin (10 μM, apical) resulted in the activation of chloride current (I_Cl_) across CF^+CFTR^ but not CF cell monolayers suggesting the activation of CFTR-mediated Cl transport by gliotoxin. **(B)** Summary of I**_Cl_** before and after gliotoxin stimulation (peak currents). Data are shown as mean values ± SE, n=3-4, * indicates p<0.05 (Wilcoxon Signed Rank Test).

The effects of gliotoxin were further studied with the Ussing assay to determine its role on CFTR-mediated chloride transport function in bronchial epithelial cells. Interestingly, addition of gliotoxin (10 µM) to the apical chamber compartment elicited a chloride secretory response in CF^+CFTR^ but not CF cell monolayers. Details of the original recordings are shown in **Figure 8A**. Gliotoxin stimulated baseline I_Cl_ from 13.4 ± 0.5 µA/cm^2^ to 24.3 ± 1.6 µA/cm^2^ (n=4, p<0.05) in CF^+CFTR^ cells. Baseline I_Cl_ did not change in CF cells and I_Cl_ averaged 5.1 ± 0.5 µA/cm^2^ before and 5.5 ± 0.5 µA/cm^2^ (n=5, p>0.05) after gliotoxin addition at similar time points (**Figure 8B**). These experiments suggested that gliotoxin is a small molecule that can transiently activate CFTR-mediated chloride secretion. The chloride secretory phase to gliotoxin occurred within 15 minutes. However, a second phase occurred approximately 30 minutes later (illustrated in **Figures 7A, B**) that was characterized by a gradual increase in I_Cl_ due to paracellular chloride movement and complete loss of electrical resistance of the cell monolayer (summarized in **Figure 7C**). These experiments suggested that gliotoxin can target CFTR Cl transport activity and destroy tight junction integrity of bronchial epithelial cells.

## Discussion

The Transwell co-culture model established in this study is a multifunctional tool for the study of direct and indirect epithelial barrier interactions with fungi. Direct mucosal addition of fungal morphotypes allows for study of fungal-epithelial cell interactions, while serosal *A. fumigatus* co-culture allows for analysis of paracrine signaling (Bogdanowicz and Lu, 2014). Such a co-culture model also allows for spatial and temporal control of a particular epithelial cell type’s interactions with different combinations of *A. fumigatus* morphotypes and secondary metabolites (Duell et al., 2011; Bogdanowicz and Lu, 2014). Similar co-culture studies have been conducted ranging from assessing *Pseudomonas aeruginosa* virulence factors to chemokine profiles from nasal epithelium following H3N2 influenza virus infection (Crabbe et al., 2014; Luukkainen et al., 2018). The CFBE41o- cell line has proven useful in understanding pathogen interactions: co-infection with both *Pseudomonas aeruginosa* and *A. fumigatus* allows for study of antagonistic effects, changes in biofilm development, etc (Reece et al., 2018). Utilization of the isogenic CFBE41o- and CFBE41o-^+CFTR^ bronchial epithelial cell model also allows for isolated analysis of differences in barrier quality or fungal-host interaction specifically due to CFTR expression. The Ussing assay has served as a gold standard for the measurement of CFTR function in drug development for cystic fibrosis. Similarly, utilization of the Ussing assay for studies of secondary metabolites has been applied for bacterial products including *Pseudomonas aeruginosa* derived pyocyanin (Schwarzer et al., 2008) and homoserine lactone (Schwarzer et al., 2010) and allows for rapid testing of potent epithelial barrier toxins produced by *A. fumigatus*, as well as the effects of these toxins in co-culture with the three *A. fumigatus* morphotypes or antifungal agents. Ussing Chamber studies also allow for collection of continuous time-point data necessary for kinetic analysis, measurements of IC50, etc. These tools have potential to be used in diverse studies of bacterial and fungal infection of both epithelial and effector cells in addition to CF.

The impacts of different *A. fumigatus* morphotypes on tight junctions and ZO-1 proteins have not been elucidated in past studies of *A. fumigatus* pathophysiology. Our study demonstrates that both CF and CF^+CFTR^ cell monolayers display significant deteriorations in airway epithelial barrier function upon exposure to conidia and germlings, with a stronger and earlier decline in transepithelial resistance noted in CF epithelial cells. Confocal microscopy for ZO-1 immunofluorescence of these epithelial cells co-cultured with conidia and germlings demonstrated disruptions in TJ barrier, as well as internalization of CO in both CF and CF^+CFTR^ bronchial epithelial cells. Hyphae resulted in the most rapid deterioration of epithelial barrier function among the three *A. fumigatus* morphotypes in both cell-lines. The *A. fumigatus*-derived virulence factor gliotoxin caused a rapid decline in transepithelial resistance at micromolar concentrations suggesting that gliotoxin can exert toxic effects on epithelial barrier function in the lung. Of note, addition of heat-killed conidia or of conidia to the basolateral surface did not cause transepithelial resistance to decline, indicating potential pathogenic mechanisms that involve the direct interaction of conidia at the cell surface and conidial growth into germlings and hyphae. These results add to the list of other bacterial and fungal toxins known to interrupt epithelial barriers. For example, toxins derived from *Pseudomonas aeruginosa* or *E. coli* are known to interrupt ZO-1 tight junction proteins and increase paracellular permeability, promoting a pro-inflammatory response similar to that of *A. fumigatus* (Sears, 2000; Soong et al., 2008).

*A. fumigatus* typically enters bronchioles as asexual spores known as conidia. Using the CFBE41o- cell line as an experimental model, we investigated the internalization of GFP-expressing conidia into bronchial epithelial cells. Our experiments demonstrated that conidia were internalized by CF bronchial cells within 6 hours. Interestingly, our data also showed that a larger number of conidia accumulated in the cytosol of CF cells (35%) when compared to the internalization in the cytosol of CFTR-corrected CF cells (18%). This observation is consistent with the corresponding effects of tight junction breakdown and deterioration of TER at the 6-hour time point and the data suggest a protective role of CFTR expression during infection with conidia. Healthy lungs typically clear inhaled conidia through innate defense mechanisms, which include mucociliary clearance (Mullins and Seaton, 1978; Bromley and Donaldson, 1996; Croft et al., 2016). Impaired clearance in immunocompromised lungs, however, has been shown to allow for adhesion of conidia to alveolar epithelial cells (AECs) via protein and carbohydrate moieties (Wasylnka and Moore, 2000; Sheppard, 2011; Chaudhary et al., 2012). Exposed basal lamina, as seen in patients prone to asthma and aspergillosis, also allows for rapid *A. fumigatus* conidial adhesion and internalization (DeHart et al., 1997). Inflammatory cytokines (i.e., IL-8), chemokines, and innate immunity proteins expressed by AECs trigger widespread inflammation (Bromley and Donaldson, 1996). Studies of AECs with non-functional CFTR demonstrated reduced killing of internalized conidia despite increased inflammatory cytokine expression; this would explain aggravated fibrosis and fungal disease progression/lung injury due to *A. fumigatus (* MOmany and Taylor, 2000 *)*.

The humid, nutrient-rich environment of alveoli allows conidia to leave dormancy and transform into germlings, which consist of the conidial cell and an elongating germ tube (Kamai et al., 2006; Ben-Ami et al., 2010). Following filamentous growth, the morphotype is multicellular hyphae rather than unicellular conidia. Hyphae are critical for tissue invasion and furthering a proinflammatory response; depending on host status, they can penetrate vascular endothelium as well (Kamai et al., 2006; Hohl and Feldmesser, 2007; Stergiopoulou et al., 2007). Hyphae are also able to release several secondary metabolites such as gliotoxin and evade macrophage phagocytosis due to their large size (Ben-Ami et al., 2010). *A. fumigatus*-secreted elastinolytic and collagenolytic enzymes further degrade alveolar tissue and compromise the integrity of the epithelial barrier (Stergiopoulou et al., 2007). Examining the effect of *A. fumigatus* on lung epithelial integrity throughout different lifecycle morphotypes is a novel approach toward understanding fungal-epithelial interactions and fungal lifecycle development. Establishing that barrier degradation progresses throughout the three predominant morphotypes of the *A. fumigatus* lifecycle can be affected by lack of normal CFTR protein in CF bronchial epithelial cells homozygous for the F508del CFTR mutation suggests the need for timely and appropriate antifungal therapy in vulnerable populations.

Gliotoxin is a major *A. fumigatus* mycotoxin (Kwon-Chung and Sugui, 2009; Bugli et al., 2014) and has been noted in lung tissue at concentrations of nearly 4000 ng/g in acute *Aspergillus* infection, consistent with the concentration tested with the Ussing assay (Lewis et al., 2005). TER data (**Figure 6D**) demonstrated a rapid decline in response to gliotoxin (at a concentration of 10 μM) in both CF and CF^+CFTR^ cells, with about 95% reduction in both groups by the 3-hour time point. Application of furosemide verified use of paracellular, rather than transcellular, pathways of chloride transport, indicative of barrier breakdown.

Our prior study investigated the appearance of gliotoxin, detected by mass spectroscopy, in supernatants of *Aspergillus fumigatus* cultured at incubator temperature *in vitro*, from conidia developing to germlings to hyphae (Patil et al., 2022). In that time course, no gliotoxin was detected in the first 31 hours, and only first appeared after 48 hours of fungal development. This course could explain the rapid trigger of a cellular response in the present study when purified gliotoxin was applied to the cells, compared to the delayed responses when viable fungal elements were applied, since gliotoxin would have been present only after hours-long fungal co-incubation.

The impacts of gliotoxin on lung epithelial integrity have not previously been studied - this study demonstrates both the immediate and rapid degradation of the epithelial barrier and notes no appreciable benefit of the CFTR-protein in reducing breakdown. The rapid degradation of the TER mirrors those of the hyphal induced damage occurring rapidly after addition to the cells. This might be explained in that the delayed response to conidia and germlings reflects the requirement of time to form hyphae and it is from the hyphae one would expect the formation of secondary metabolites such as gliotoxin. As a secondary metabolite of *A. fumigatus* and immunosuppressive agent itself, gliotoxin has been noted to target leukocyte migration and superoxide production (Kamei and Watanabe, 2005; Kupfahl et al., 2006). Our studies demonstrate that it may also facilitate fungal invasion into healthy lung tissue and thus evasion of immune cell attack. In order to study the direct involvement of gliotoxin throughout the three life cycle morphotypes, future studies may be conducted comparing gliotoxin-producing vs. gliotoxin-null mutant *A. fumigatus* conidia, germlings, and hyphae under a similar protocol. Gliotoxin is one of over 200 secondary metabolites produced by *A. fumigatus* and resulted in strong barrier breakdown; similar transepithelial resistance experiments should be conducted on other *A. fumigatus* metabolites such as fumagillin, helvolic acid, and fumitremorgin A.

As demonstrated in this study*, A. fumigatus* can cause damage to lung epithelial barrier throughout different stages of its lifecycle, notably conidia, germlings, and hyphae. Likewise, its major virulence factor gliotoxin can cause similar rapid degradation of barrier function, aggravating injury. The Transwell co-culture model proves a useful method for the study of apical interactions between epithelial cells, pathogens, and pathogen metabolites over time. Notably, much consideration has been given in the literature to the effect of excessive mucus in CF airways in producing aberrant responses to microbes in such airways. Our study, in contrast, appears to indicate that there are intrinsic differences in the CFTR-negative cells themselves, which would explain or contribute to the dysfunctional responses to microbes, at least to fungal pathogens. Acute addition of gliotoxin caused a chloride secretory response in CF^+CFTR^ cells but not CF cells, suggesting that gliotoxin specifically targets the CFTR-mediated Cl transport and not the calcium-activated chloride conductance that is present in this CF cell line. Further studies are needed to understand the underlying mechanism for the activation of normal CFTR function by gliotoxin.

## Data availability statement

The raw data supporting the conclusion of this article will be made available by the authors without undue reservations.

## Author contributions

BI: conceptualization, experimentation, data analysis, writing. HF: supervision, resources, data analysis, visualization, acquisition. TM: conceptualization, supervision, writing. GH: experimentation, data analysis, writing. KC: conceptualization, supervision, writing. GS: execution, data analysis, writing. JF: experimentation, data analysis, writing, DS: conceptualization, supervision, resources, writing, financial support. All authors contributed to the article and approved the submitted version.
