# Supplementary material for: Protective role of CFTR during fungal infection of cystic fibrosis bronchial epithelial cells with Aspergillus fumigatus
Source: Front Cell Infect Microbiol. 2023 Aug 23;13:1196581. doi: 10.3389/fcimb.2023.1196581 (PMC10482090; doi:10.3389/fcimb.2023.1196581)
Supplement: Supplementary file 1 [file DataSheet_1.docx]

Supplementary Material

Protective role of CFTR during fungal infection of cystic fibrosis bronchial epithelial cells with *Aspergillus fumigatus*

**Beate Illek*^1,a^, Horst Fischer^1^, Terry E. Machen^2^, Gopika Hari^1^, Karl V. Clemons^3,4^, Gabriele Sass^3^, Jose A. G. Ferreira^3,4,b^, David A. Stevens^3,4^**

*** Correspondence:** Beate Illek: [beillek@health.ucsd.edu](mailto:beillek@health.ucsd.edu)

# Supplementary Data.

**Supplementary Figure 1.**

The epithelial co-culture model was used to study the potency of non-viable *A. fumigatus* conidia on epithelial barrier function by exposing the apical (upper) compartment of the Transwell cell culture insert to heat-killed Af. conidia for a period of 24 hours. *A. fumigatus* conidia were heat-killed by incubation at 65 °C until no viable conidia were detected and added to the apical at a final concentration of 5,000 CO/~10^6^ cells. Transepithelial electrical resistances (TER) were monitored by an epithelial volt ohmmeter at time = 0, 3, 6, 12, and 24 hours. Average TER values of non-treated (open circles) vs. conidia-treated are plotted for **A.** CFBE41o- (CF; red circles) or **B.** CFTR-expressing CFBE41o- (CF^+CFTR^; black circles) cell monolayers. TER values were not different between treated and time-matched non-treated controls (P>0.05; Welch’s t-test) and not affected by treatment at all time points within the CF or CF^+CFTR^ group (P>0.05; Repeated Measures ANOVA on Ranks). **C.** Transepithelial resistance values in response to apical exposure to heat-killed Af. conidia were normalized to non-treated CF or CF^+CFTR^ cultures at t = 0, 3, 6, 9, 12, and 24 hours. Normalized TER values from same time points were similar between CF (red circles) and CF^+CFTR^ (black circles) (P>0.05; Welch’s t-test). Data are shown as mean values ± SD from n=8 experiments.


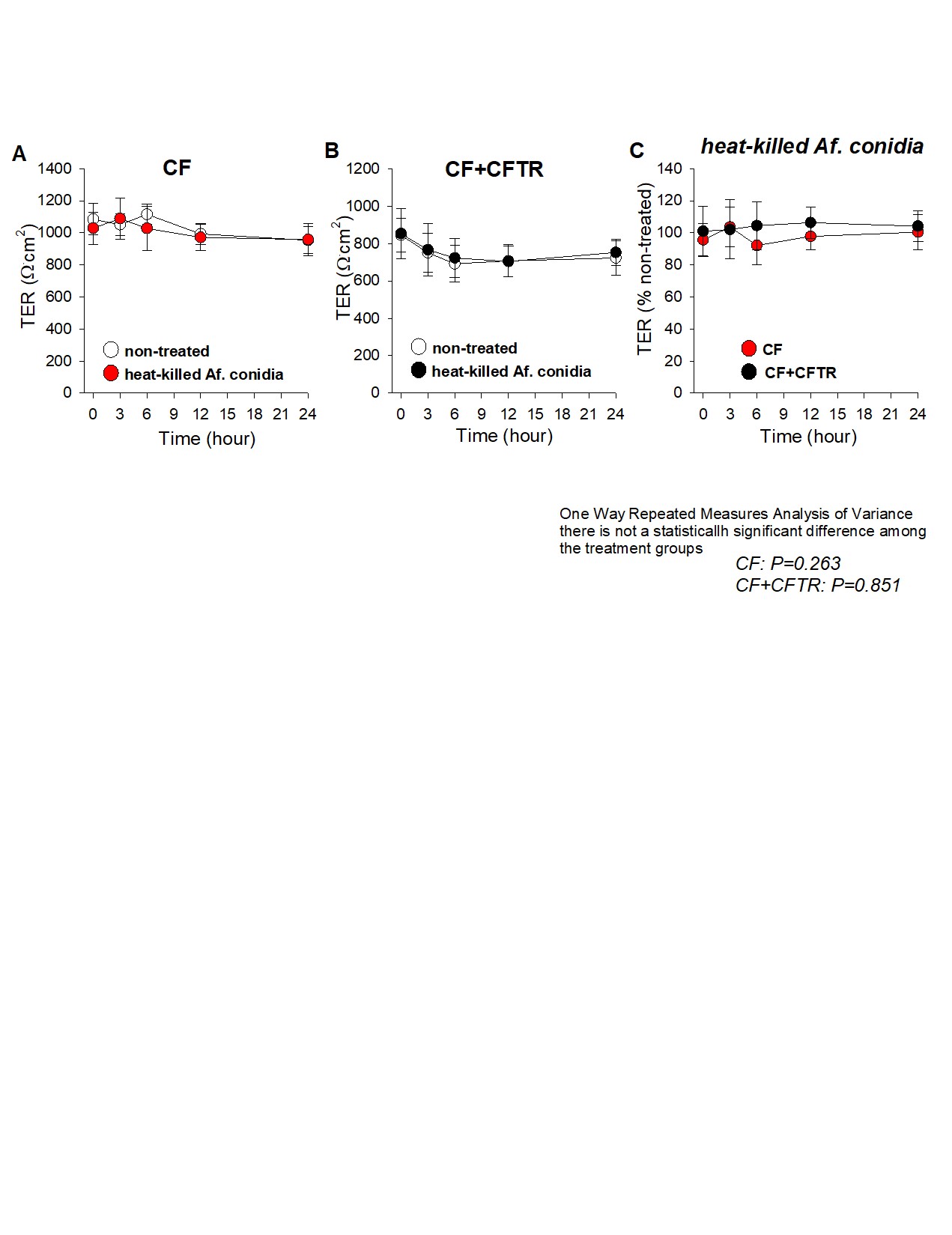


**Supplementary Figure 2.**

The epithelial coculture model was used to study side-specific effects of *A. fumigatus* conidia on epithelial barrier function by exposing the serosal side of the bronchial epithelial cell monolayer to conidia. Af. conidia (5,000 CO) were added to the serosal (bottom) compartment of the Transwell cell culture insert for a period of 24 hours. Transepithelial electrical resistance (TER) values were monitored by an epithelial volt ohmmeter at time = 0, 3, 6, 9, 12, and 24 hours. Average TER values ± SD are plotted for non-treated (open circles) and conidia-treated **A.** CFBE41o- (CF, red circles) or **B.** CFTR-expressing CFBE41o- (CF^+CFTR^; black circles) cell monolayers. TER values were not different between treated and time-matched non-treated controls (P>0.05; Welch’s t-test) and not affected by treatment at all time points within the CF or CF^+CFTR^ group (P>0.05; Repeated Measures ANOVA on Ranks). **C.** Transepithelial resistance values in response to serosal exposure to Af. conidia were normalized to non-treated CF or CF^+CFTR^ cultures at t = 0, 3, 6, 9, 12, and 24 hours. Normalized TER values from same time points were similar between CF (red circles) and CF^+CFTR^ (black circles) (P>0.05; Welch’s t-test). Data are shown as mean values ± SD from n=8 experiments.

This set of experiments demonstrated that exposure of the basolateral cell membrane to A. fumigatus conidia does not lead to epithelial barrier breakdown. In contrast, exposure of the apical cell membrane (upper chamber) resulted in a complete loss of TER (plotted in Figure 6A) suggesting the involvement of a specific signaling mechanism in the apical membrane. However, it is also conceivable that the small pore size of the Transwell insert (0.45 μm) could have limited the cellular access of Af. conidia from the basolateral cell membrane.


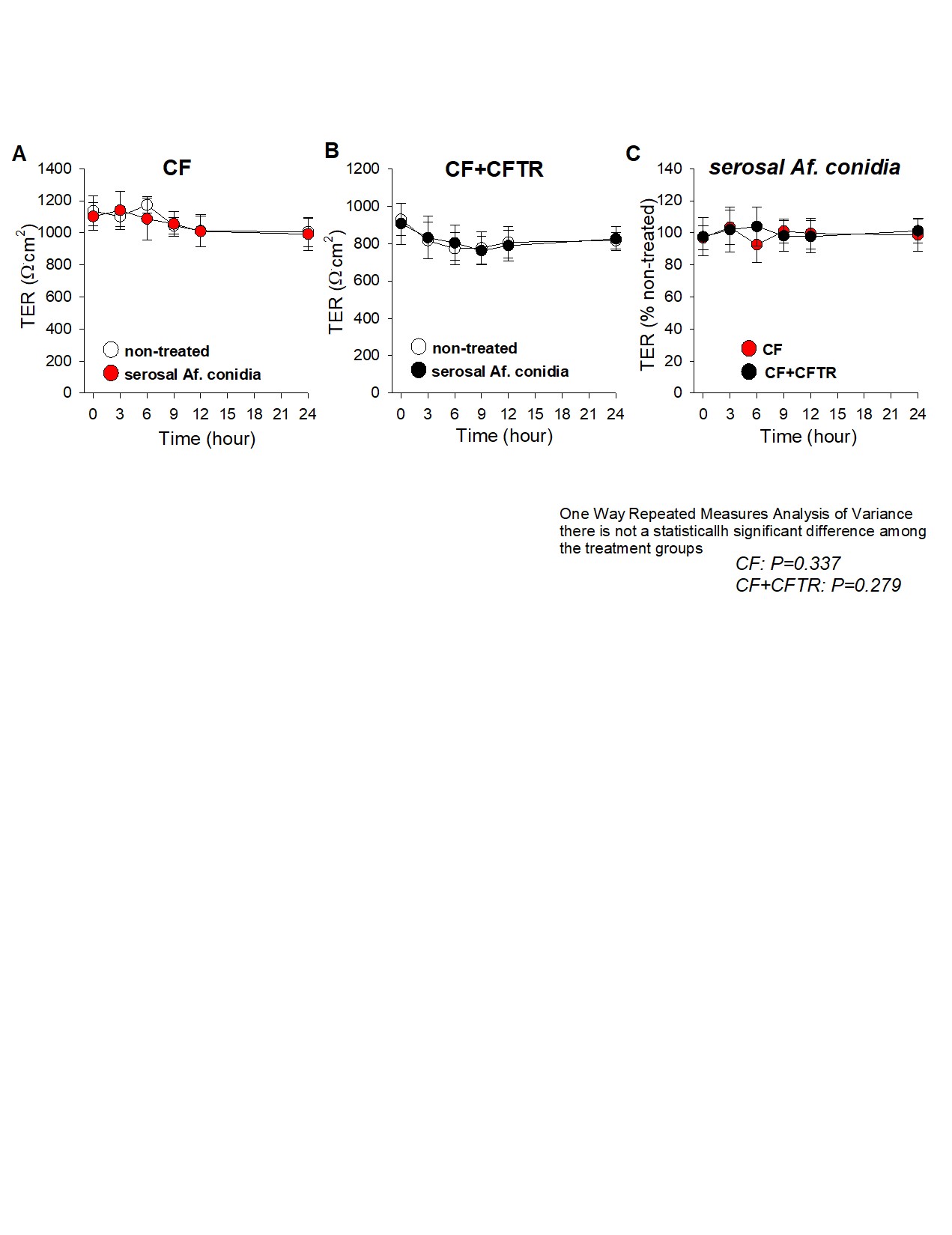


**
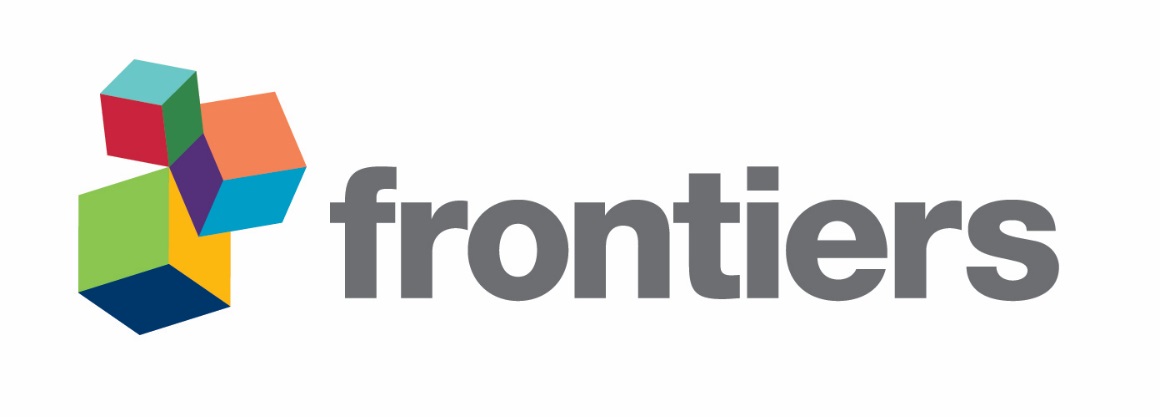
**
